# Co-occurring nematodes and bacteria in submarine canyon sediments

**DOI:** 10.7717/peerj.5396

**Published:** 2018-07-31

**Authors:** Jadwiga Rzeznik-Orignac, Antoine Puisay, Evelyne Derelle, Erwan Peru, Nadine Le Bris, Pierre E. Galand

**Affiliations:** 1 Laboratoire d’Ecogéochimie des Environnements Benthiques, LECOB, Sorbonne Université, CNRS, Banyuls-sur-Mer, France; 2 Criobe, Laboratoire d’Excellence “Corail”, PSL Research University: EPHE-UPVD-CNRS, Papetoai, French Polynesia; 3 Laboratoire de Biologie Intégrative des Organismes Marins, Sorbonne Université, CNRS, Banyuls-sur-Mer, France; 4 LEMAR UMR 6539 CNRS/UBO/IRD/Ifremer, IUEM, Plouzané, France

**Keywords:** Bacteria, Meiofauna, Canyon Lacaze-Duthiers, Metabarcording, Molecular approach, Mediterranean Sea, Nematodes, Marine biodiversity, 18S, 16S

## Abstract

In submarine canyon sediments, bacteria and nematodes dominate the benthic biomass and play a key role in nutrient cycling and energy transfer. The diversity of these communities remains, however, poorly studied. This work aims at describing the composition of bacteria and nematode communities in the Lacaze-Duthiers submarine canyon in the north-western Mediterranean Sea. We targeted three sediment depths for two consecutive years and investigated the communities using nuclear markers (18S rRNA and 16S rRNA genes). High throughput sequencing combined to maximal information coefficient (MIC) statistical analysis allowed us to identify, for the first time, at the same small scale, the community structures and the co-occurrence of nematodes and bacteria Operational Taxonomic Units across the sediment cores. The associations detected by MIC revealed marked patterns of co-occurrences between the bacteria and nematodes in the sediment of the canyon and could be linked to the ecological requirements of individual bacteria and nematodes. For the bacterial community, *Delta*- and *Gammaproteobacteria* sequences were the most abundant, as seen in some canyons earlier, although *Acidobacteria*, *Actinobacteria* and *Planctomycetes* have been prevalent in other canyon sediments. The 20 identified nematode genera included bacteria feeders as *Terschellingia*, *Eubostrichus*, *Geomonhystera*, *Desmoscolex* and *Leptolaimus.* The present study provides new data on the diversity of bacterial and nematodes communities in the Lacaze-Duthiers canyon and further highlights the importance of small-scale sampling for an accurate vision of deep-sea communities.

## Introduction

Submarine canyons represent deep incisions in the continental slope, that enlarge the heterogeneity of deep-sea habitats on the shelf break and generate biodiversity (Levin et al., 2010). Canyons are widespread, and many of them were described to actively transport sediments and organic debris directly from shallow waters to the deep-sea (De Leo et al., 2010). In the Gulf of Lion (the north-western Mediterranean Sea), water transport driven by density contrast between surface and deeper waters occurs in winter through the formation of dense surface shelf waters by cold winds (Canals et al., 2006). This cascading phenomenon, and more generally the hydrodynamic features (including storm downwelling and eddies) promoting a fast and episodic transfer of organic material from the shelf to the deep sea, convert canyons into hotspots of benthic biomass (De Leo et al., 2010).

In canyon sediments, bacteria dominate benthic biomass and play a key role in nutrient cycling and energy transfer (Danovaro et al., 2010; Pusceddu et al., 2012). Our knowledge of the bacteria dwelling submarine canyon sediments is, however, limited. Early studies based on quantitative approaches showed that canyon sediments had higher densities of bacterial abundance compared to densities found in other deep-sea sediments at similar depth (Danovaro et al., 1999). The few existing qualitative studies are limited to descriptions at the phylum or class level and show high proportion of *Acidobacteria*, *Actinobacteria* and *Gammaproteobacteria* in surface sediments at the mouth of a canyon in the Eastern Mediterranean (Polymenakou et al., 2009) and *Gammaproteobacteria* and *Planctomycetes* in the Monterrey Canyon (Goffredi & Orphan, 2010). A recent study, however, showed the dominance of *Gamma*- and *Deltaproteobacteria* in canyons in the Gulf of Mexico and off the coast of California (Crespo-Medina et al., 2016; Harrison et al., 2018). A study of the structure of bacterial community by fingerprinting techniques showed differences in composition in sediment surface layers between canyons in the north-eastern Atlantic Ocean but the identity of the bacteria was not reported (Amaro et al., 2009).

The meiofauna, that is, benthic invertebrates (<1 mm in size), also plays a major role in the ecology of canyon sediments (Danovaro et al., 2010; Gambi, Lampadariou & Danovaro, 2010). Meiofauna is very abundant, accounts for the main part of the metazoan diversity and plays an important role in the maintenance of marine biogeochemical cycle (Giere, 2009). Typically, meiofaunal abundance and biomass are higher in canyon sediments compared to other habitats at comparable depths (Rosli et al., 2016). This is attributed to the accumulation of organic matter in canyons which may stimulate their growth.

Free-living nematodes are among the most abundant and diverse metazoans of meiofaunal taxa in deep-sea sediments (Vincx et al., 1994). The nematodes often show an aggregated vertical distribution within sediments that is attributed to different driving factors. For example, in coastal sediments, the tidal cycle is the most important factor that affects the chemical and physical micro-environment structure and drives the distribution of nematodes (Harris, 1972; Joint, Gee & Warwick, 1982; Steyaert et al., 2001, 2003). In more stable sub-tidal and deep-sea habitats biogenic factors, such as food supply or animal interactions, gain importance. Among them, the most relevant and ubiquitous biogenic factors that are correlated to nematode vertical distribution are the abundance of detritus, protists and bacteria (Giere, 2009; Ingels et al., 2013). However, oxygen penetration and the occurrence of sulfide are thought to be some of the important regulating factors explaining vertical distribution patterns (Steyaert et al., 2003).

While there is a general paucity of data on co-occurrence of nematodes and bacteria in deep-sea sediments (Ingels et al., 2011), insight into the small-scale (corresponding to about one cm^3^ of sediment) community structure is required to understand the biogeochemical and ecological processes that play key functional roles in marine sediments. Previous works addressing the question with quantitative tools reported contrasting results. High numbers and biomass of meiofauna correlated with low numbers and biomass of bacteria in sandy beach sediments (Meyer-Reil & Faubel, 1980), absence of correlation between meiofauna and bacterial biomass in the deep Aegean Sea (Danovaro et al., 1995), but strong correlation between meiofaunal densities and viable bacterial number in the deep part of Gulf of Lion (De Bovée, Guidi & Soyer, 1990) were also observed. Recent work that targeted more precisely some ecosystem processes showed that ecological interactions between meiofauna and benthic bacteria are important in the ecology of sediments (Thurber, 2015). Nitrogen cycling may be regulated by these interactions as shown by the stimulating effect that meiofauna has on nitrifying and denitrifying bacteria (Bonaglia et al., 2014). Meiofauna may also enhance the mineralization of organic matter by stimulating the activity of sediment bacterial community (Nascimento, Näslund & Elmgren, 2012), while higher abundances of meiofauna may decrease the microbial mineralization of naphthalene by modifying the bacterial community composition through bacterial grazing by metazoans (Näslund, Nascimento & Gunnarsson, 2010).

The present study had two complementary purposes: to characterize the diversity of the nematodes and the bacteria in the sediment of the Lacaze-Duthiers submarine canyon, and to investigate patterns of co-occurrence between individual bacteria and nematode taxa. We studied the communities by sequencing the 18S rRNA and 16S rRNA genes to describe eukaryotic and bacterial communities respectively.

## Materials and Methods

### Sample collection and processing

Sampling was carried out in the head of the Lacaze-Duthiers canyon in the Gulf of Lion (NW Mediterranean, 42°32′26″N, 03°25′9″E) (Fig. S1). Three sediment cores (six cm of diameter and 18 cm of height) were collected in September 2011 and three in July 2012 at 525 m depth using a multiple mini corer (OSIL, Havant, UK) deployed from the research vessel Minibex. The remotely operated underwater vehicle used for exploration and experiment deployment in this area of the canyon hosting abundant cold-water coral assemblages on rocky outcrops allowed to precisely position the corer and sample the sediment in the same area for both sampling campaigns. On board, each core was immediately cut horizontally and samples were gathered from three different layers: 0–1, 3–5 and 16–18 cm. The sediment is a mud containing more than 90% of silt. The subdivision into three different layers, 0–1, 3–5 and 16–18 cm, is based on the on board visual analysis of sediment cores that allowed us to develop a sampling protocol used throughout the series of sampling campaigns. We wanted contrasted types of sediments so we chose: the 0–1 cm deep layer that represents the surface sediment layer, the 3–5 cm layer that corresponds to changes in sediment color and usually contains high nematode abundances, and the 16–18 cm layer that represents the dark completely anoxic sediment layer. All material was carefully washed with ethanol before cutting each layer to prevent any contamination. Sediment from each layer was gently homogenized. Sediment samples (about one g wet weight) dedicated to bacterial analysis were immediately frozen at −80 °C and the ones for nematodes analysis were stored at 10 °C in order to preserve (3 h) living animals until return to the shore-based laboratory of the Marine Station of Banyuls). In the laboratory, the sediment samples dedicated to analyze the nematodes were immediately processed by centrifugation in Ludox solution to separate the metazoans from the sediment (Heip, Vincx & Vranken, 1985). Then, the extracted meiofauna was frozen at −80 °C until DNA extraction.

### DNA extraction

DNA for nematode analysis was extracted using the CTAB method with manual grinding (Winnepenninckx, Backeljau & De Wachter, 1993). The protocol applied to analyze of meiofauna was previously described in Rzeznik-Orignac et al. (2017). Eukaryotic 18S rRNA genes were amplified using universal primers Euk528 (5′-CCGCGGTAATTCCAGCTC-3′) and R18 (5′-CGTTATCGGAATTAACCAGAC-3′). Eukaryotic sequences could only be obtained from two cores (1 in 2011 and 1 in 2012) and these two cores were chosen for prokaryotic sequencing. Bacterial DNA was extracted directly from one g of sediment using the PowerSoil kit (MoBio, Carlsbad, CA, USA) following the manufacturer’s instructions. Bacterial 16S rRNA genes were amplified using primers 28F (5′-TTTGATCNTGGCTCAG-3′) and 519 R (5′-GTNTTACNGCGGCKGCTG-3′). Sequence data were deposited in the GenBank/EMBL/DDBJ short-read archive as submission ID: SRP127023 and SRP137819 (for 16S and 18S respectively).

### Pyrosequencing and sequence analysis

The 16S and 18S rRNA genes were sequenced with a Roche 454 FLX using Titanium reagents by a commercial laboratory (Research and Testing Laboratory, Lubbock, TX, USA). The quality of the sequences was controlled by removing all reads that had a mismatch with the 16S and 18S rRNA genes primers, contained ambiguous nucleotides (N) or that were less than 230 nucleotides long after the forward primer. Reads having ≥3% of bases with Phred values <27 (0.2% per-base error probability) were removed. This is recommended to ensure that, when clustering at 97%, the influence of erroneous reads is minimized (Huse et al., 2010; Kunin et al., 2010). Sequences were then de-replicated and clustered at a 97% threshold using Uclust algorithm (Edgar, 2010). Read quality filtering and length trimming, de-replication, clustering at 97% sequence identity, taxonomic classification and dataset partitioning based on barcodes were conducted with Pyrotagger (Kunin & Hugenholtz, 2010). Sequences from each Operational Taxonomic Units (OTU) were classified by comparison to the Greengenes database (DeSantis et al., 2006) and Silva database for bacteria and eukaryotes respectively. The taxonomic affiliations of the most abundant OTUs (>1% of the sequences) were further verified against sequences from the National Center for Biotechnology Information databases using BLAST (Altschul et al., 1997). For bacteria, datasets were randomly resampled down to 759 sequences per sample using daisychopper (Gilbert et al., 2009), which represent the number of sequences in the smaller sample, for an unbiased comparison of diversity.

### Data analysis

We calculated richness and the Shannon diversity index for all communities with the program PAST (Hammer, Harper & Ryan, 2001). The relationship between communities within the domain bacteria and within the eukaryotes was estimated by cluster analysis based on Bray–Curtis dissimilarity indexes calculated between communities and used to construct a dendrogram with an unweighted pair group method with arithmetic mean. For bacteria, we used abundance data whereas we used presence–absence data for nematodes due to more variable 18S rRNA gene copy number between species.

The co-occurrence of bacterial and nematode OTUs was analyzed through MINE statistics by calculating the maximal information coefficient (MIC) between each pair of OTUs (Reshef et al., 2011). MIC captures associations between data and provides a score that represents the strength of a relationship between data pairs. The matrix of MIC values >0.5 and corresponding to positive linear correlations was used with Cytoscape 3.1.1 to visualize the network of associations (Smoot et al., 2011). In these visualizations, OTUs are represented as nodes and are connected by lines that are proportional in length to the MIC value. The force-directed layout was edge-weighted by the MIC value. The presence of sub-networks within the main network was assessed with the CytoCluster application using the graph clustering algorithms HC-PIN with default options (Wang et al., 2011).

## Results

### Community composition

**Figure 1:**
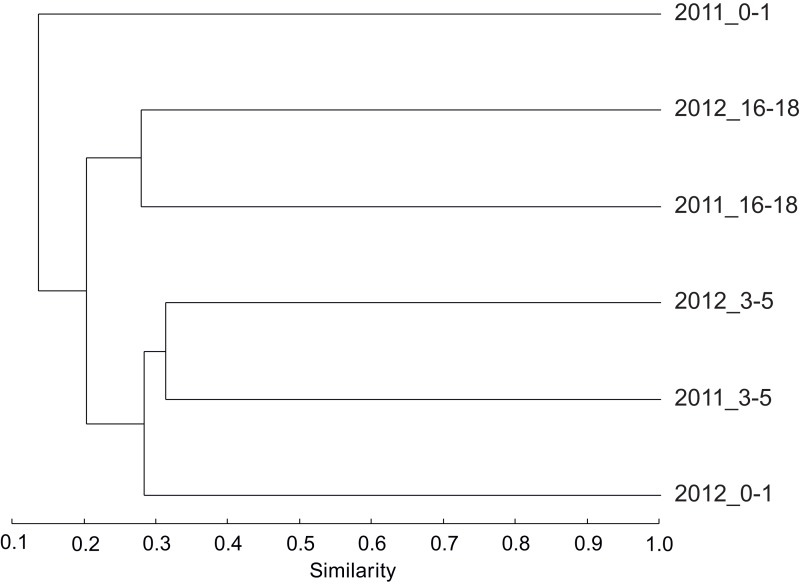
The similarity between bacterial communities. Dendrogram based on the Bray–Curtis index showing the similarity between bacterial communities sampled in 2011 and 2012 from sediment surface (0–1 cm), intermediate (3–5 cm) and deep layers (16–18 cm) in the Lacaze-Duthiers submarine canyon.

For bacteria, a cluster analysis of community composition showed that communities changed with depth for both sampling years (Fig. 1). The two deeper layer samples grouped together, separated from the intermediate layer that was again different from the surface layer. The surface layer communities appeared to be the most variable in composition, as the samples from 2011 to 2012 did not group together. The surface layer communities had in average higher richness (number of OTUs) and diversity (Shannon H) than deeper samples (Table 1).

**Table 1:** Bacterial sequences.

	Sequences	OTUs	Shannon_H
2012_0–1	786	417	5.7
2012_3–5	793	409	5.6
2012_16–18	1,160	390	5.5
2011_0–1	1,887	513	6.0
2011_3–5	759	409	5.7
2011_16–18	1,061	413	5.6

**Note:**

Number of bacterial sequences, number of OTUs and Shannon index obtained for bacterial communities sampled in 2011 and 2012 from sediment surface (0–1 cm), intermediate (3–5 cm) and deep layers (16–18 cm).

**Figure 2:**
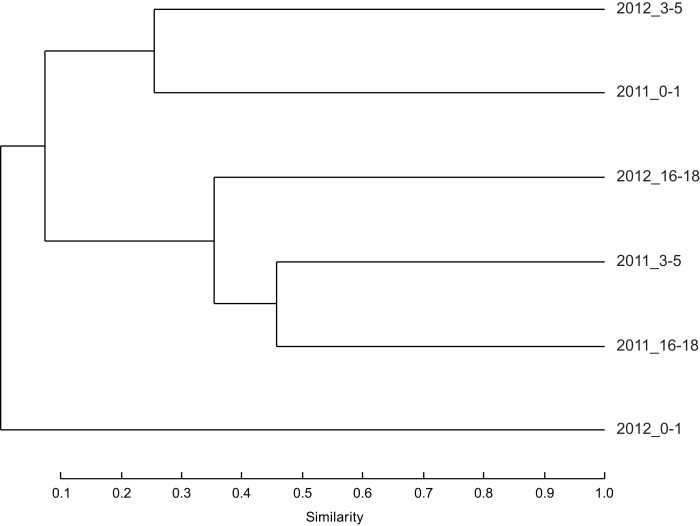
The similarity between nematode communities. Dendrogram based on the Bray–Curtis index showing the similarity between nematode communities sampled in 2011 and 2012 from sediment surface (0–1 cm), intermediate (3–5 cm) and deep layers (16–18 cm) in the Lacaze-Duthiers submarine canyon.

For nematodes, a cluster analysis (Fig. 2) of community composition did not show any clear grouping either by depth layers or by sampling periods. The surface layers communities appear to be more variable in composition than intermediate and deep layers communities. Only, two deep layers were gathered into the same cluster. In regard to the OTUs richness, no trend could be observed either by deep layers or by year (Table 2).

**Table 2:** Nematode sequences.

	Sequences	OTUs	Shannon_H
2012_0–1	196	28	2.72
2012_3–5	6,196	53	1.70
2012_16–18	1,627	12	1.10
2011_0–1	3,019	35	1.31
2011_3–5	701	15	1.63
2011_16–18	945	35	2.30

**Note:**

Number of nematodes’ sequences, number of OTUs and Shannon index for nematode communities sampled in 2011 and 2012 from sediment surface (0–1 cm), intermediate (3–5 cm) and deep layers (16–18 cm).

### Co-occurrence of bacteria and nematodes

**Figure 3:**
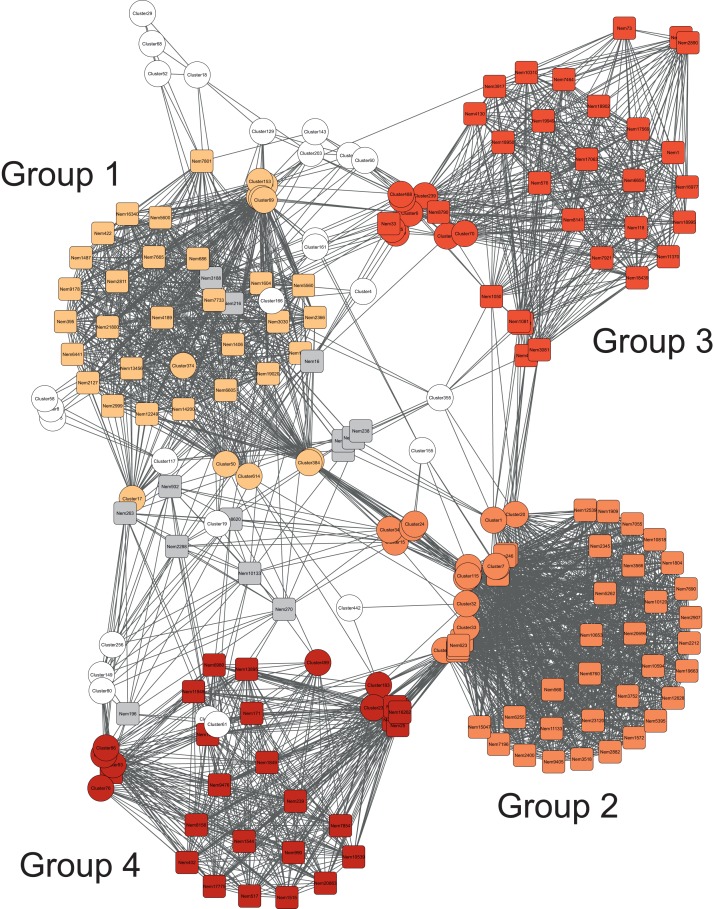
Association network of the nematode and bacteria communities. Association network of the nematode (squares) and bacteria (circles) communities in which nodes correspond to OTUs and edges correspond to relationships calculated with the MIC statistics. Colors highlight the four different subnetworks (groups) defined with the HC-PIN algorithm (see Methods).

**Figure 4:**
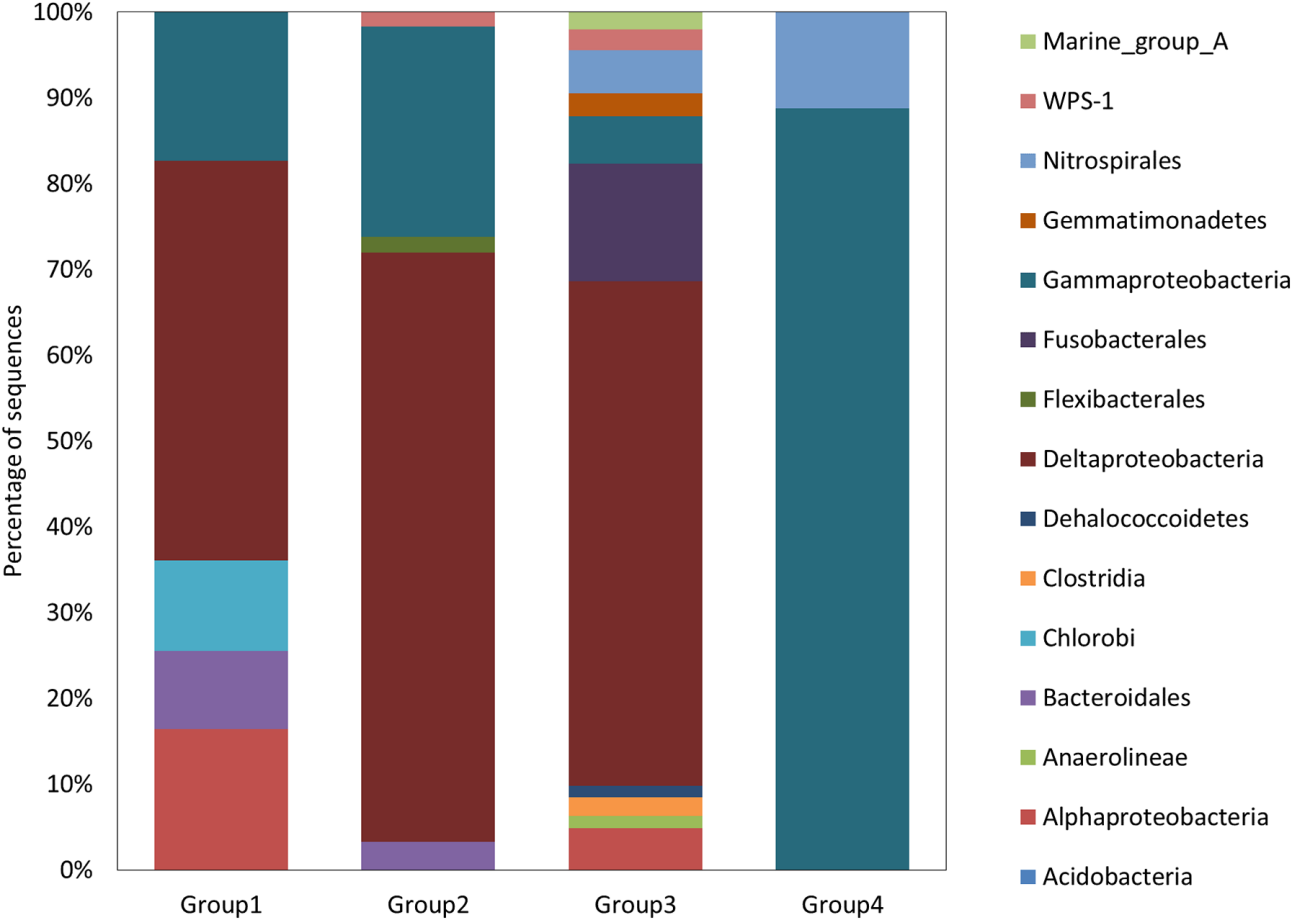
Bacterial diversity. Relative proportions of the bacterial phylum or class found in the four subnetworks.

**Figure 5:**
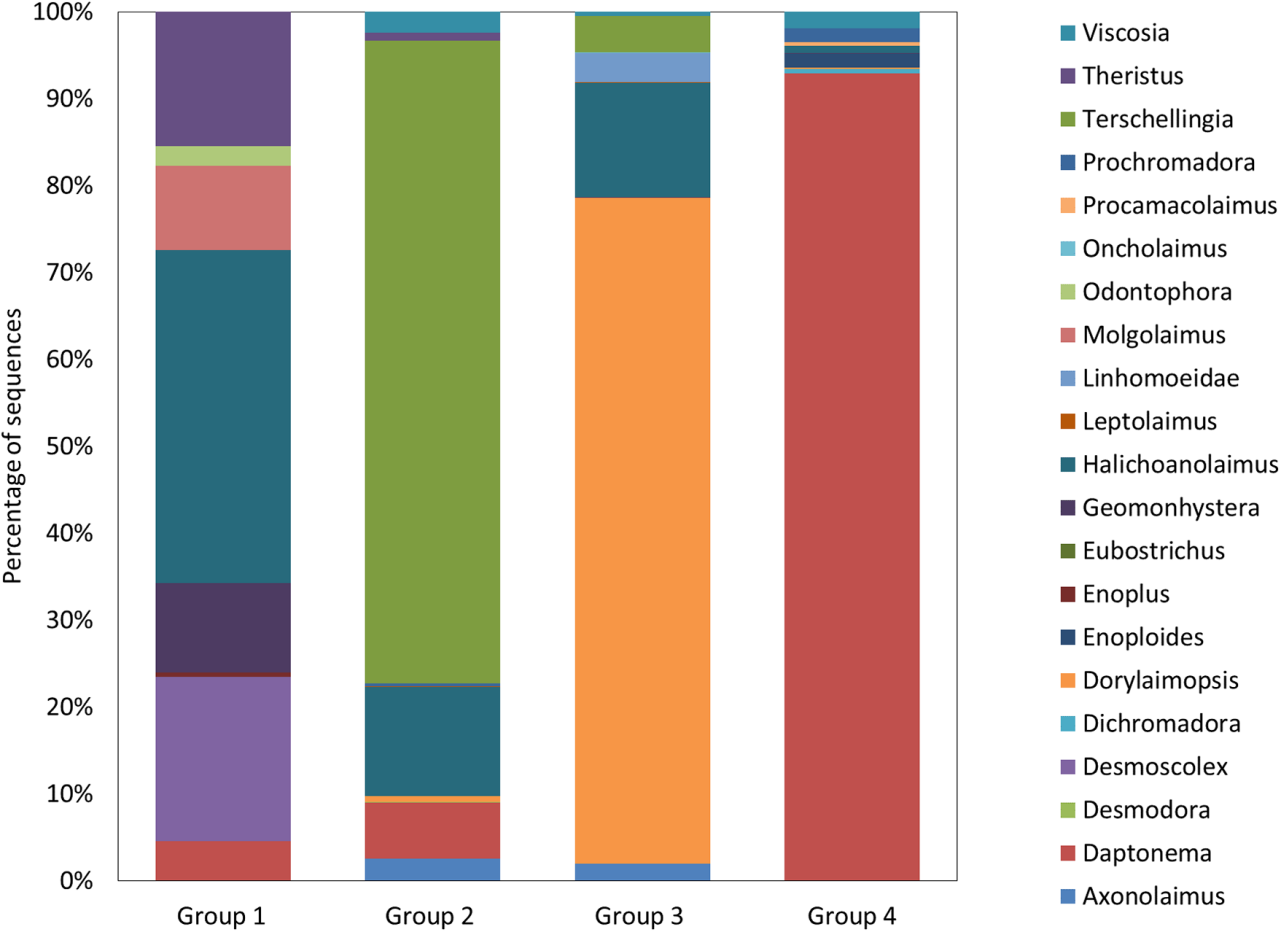
Nematode diversity. Relative proportions of the nematodes’ genera found in the four subnetworks.

The association network constructed from the MIC values showed that bacteria and nematode OTUs separated in four different subnetworks. Group 1, with highest modularity (i.e., densest connections between nodes) (Fig. 3), contained bacteria belonging to *Deltaproteobacteria*, *Gammaproteobacteria*, *Alphaproteobacteria*, *Bacteroidales* and *Chlorobi* (Fig. 4). *Chlorobi* (OTU 188) was only detected in group 1 and the OTU was similar to sequences found in anoxic sediments. All OTUs had only low similarity (<93%) with cultured microorganisms. This subnetwork contained eight nematodes genera belonging to non-selected deposit-feeders (*Daptonema*, *Theristus* and *Odontophora*), predators (*Enoplus* and *Halichoanolaimus*), selective deposit-feeders (bacteria feeders) (*Desmoscolex* and *Geomonhystera*), and one epigrowth feeder (*Molgolaimus*) (Fig. 5). Among these eight genera found in the subnetwork, a large proportion of sequences came from *Halichoanolaimus*.

The second subnetwork, group 2, contained a large majority of bacterial sequences affiliated to *Deltaproteobacteria* followed by *Gammaproteobacteria* (Fig. 4). Among *Deltaproteobacteria*, 19% of the sequences were identified as belonging to potential sulfate reducers. The group contained 10 nematode genera among them: epigrowth feeders (*Desmodora*, *Dorylaimopsis* and *Prochromadora*), non-selective deposit-feeders (*Axonolaimus*, *Daptonema* and *Theristus*) as well as bacteria feeders but they were different from those detected in the first subnetwork (*Terschellingia* and *Leptolaimus*), and predators (*Halichoanolaimus* and *Viscosia*). A large proportion of nematodes’ sequences were affiliate to *Terschellingia* (Fig. 5).

The next subnetwork, group 3, contained mostly sequences from *Deltaproteobacteria* but these were not the same as the ones found in group 2. Less abundant groups such as *Fusobacterales* were found only in group 3 (Fig. 4). The main *Fusobacterales* OTU was similar (98%) to an anaerobic strain of *Propionigenium maris* able to ferment carbohydrates (Janssen & Liesack, 1995). The most abundant *Deltaproteobacteria* was distantly related (90%) to the sulfate reducer *Desulfosarcina variabilis*. Group 3 also contained sequences affiliated to *Nitrospirales*. In this subnetwork, eight nematode genera were found; among them were bacteria feeders (*Terschellingia*, *Leptolaimus* and *Geomonhystera*) and predators (*Oncholaimus*, *Halichoanolaimus* and *Viscosia*) (Fig. 5). The subnetwork contained only one non-selective deposit-feeder (*Axonolaimus*) and one epigrowth feeder (*Dorylaimopsis*). The network contained mostly the sequences of *Dorylaimopsis*.

The last subnetwork, group 4, was characterized by the presence of sequences belonging to the genus *Nitrosospira* (Fig. 4) that were similar (96%) to strains of ammonia oxidizers (Purkhold, 2003). Many group 4 sequences belonged to *Gammaproteobacteria*, of which some were possible sulfur-oxidizing bacteria (OTU 76). Regarding the nematodes, this subnetwork contained 11 genera: four genera were classified as epigrowth feeders (*Dichromadora*, *Prochromadora*, *Dorylaimopsis*, *Procamacolaimus*), three genera as predators (*Halichoanolaimus*, *Viscosia* and *Enoploides*), two as bacteria feeders (*Eubostrichus* and *Geomonhystera*) and two as non-selective deposit-feeders (*Daptonema* and *Odontophora*) (Fig. 5). This sub network had the highest proportion of sequences affiliated to *Daptonema*.

## Discussion

In this paper, we present the first detailed description of the bacterial and nematodes diversity in a submarine canyon based on high throughput sequencing analysis. Earlier studies have shown a higher biomass of meiofauna and bacteria along the axes of canyon sediments compared to other seafloor areas at similar depth (Danovaro et al., 1999) but the detailed composition of the communities has never been reported and possible associations between nematodes and bacteria are not known. We found that at the class level, *Delta*- and *Gammaproteobacteria* sequences were the most abundant. This result is in accordance with recent studies showing the dominance of *Delta*-, *Gamma*- *and Epsilonproteobacteria* in a canyon in the Gulf of Mexico (Crespo-Medina et al., 2016) and *Delta*- and *Gammaproteobacteria* off the coast of California (Harrison et al., 2018). It differs, however, from earlier studies that showed sediments characterized by *Acidobacteria*, *Actinobacteria* and *Planctomycetes* in the Eastern Mediterranean (Polymenakou et al., 2009) and in the Monterrey Canyon (Goffredi & Orphan, 2010). At the OTU level, most sequences were only distantly related to known organisms but the similarity to some cultured strains suggested the presence of bacteria potentially associated to the sulfur and nitrogen cycles. In these sediments, bacteria showed a clear stratification with depth as generally described along redox gradient in marine sediments (Jorgensen et al., 2012).

The number of nematodes genera encountered in our study (20 genera) is comparable to those reported for the north-western Mediterranean (Catalan margin), including two canyons (Cap de Creus and Lacaze-Duthiers), and based on analysis of 12 stations (Danovaro et al., 2009). The nematode community of the Lacaze-Duthiers canyon includes essentially nematodes already encountered in deep-sea sediments (Soetaert & Heip, 1995; Danovaro et al., 2009; Hasemann & Soltwedel, 2011; Tchesunov, Ingels & Popova, 2012; Sevastou et al., 2013). We were able to detect the presence of four trophic types, among them: (1) bacteria feeders as *Terschellingia*, *Eubostrichus*, *Geomonhystera*, *Desmoscolex* and *Leptolaimus*, (2) non-selective deposit-feeders as *Daptonema*, *Theristus*, *Axonolaimus*, *Odontophora*, (3) epistrate feeders as *Dorylaimopsis*, *Desmodora*, *Molgolaimus*, *Prochromadora* and (4) predators as *Halichoanolaimus*, *Enoploides*, *Enoplus*, *Viscosia* and *Oncholaimus*.

The separation with depth was not as clear for nematodes as for bacteria community. The difference may be due to the method that we used. We chose to analyze the same quantity of sediment (one g) for both eukaryote and bacteria communities to be able to make a direct comparison between the two domains of life. Such a sample volume, which is classically used to cover bacterial diversity in sediments, may be too small to cover the full diversity of larger meiofauna organisms. Some nematodes may not have been present in the gram of sediment and it may have created artificial differences between samples. A larger volume of sediment may give a higher resolution of the meiofauna diversity. We could also have compared community composition between replicate samples, but, unfortunately, it was not possible in the present study. However, the fact that communities grouped with depth 2 years in row is a good indication that the sampling gave a fair representation of the biological conditions in situ.

Despite the different patterns of depth stratification showed by nematodes and bacteria, we were able to identify four groups of bacterial OTUs co-occurring with nematode OTUs by calculating correlations and conducting network analysis. Different combinations of bacteria and nematodes OTUs characterize each of the four groups identified. Group 4 appears to contain species able to cope with the presence of oxygen since we detected *Nitrospirales* known as potential nitrifiers that gain energy via conversion of nitrite to nitrate and live in aerobic environment (Bock & Wagner, 2006). The presence of possible sulfur-oxidizing bacteria is also an indication that group 4 organisms are living in an aerobic environment. Among nematodes, *Daptonema* OTUs are the most abundant in this group. *Daptonema* is considered as a non-selective deposit feeder. These nematodes are opportunistic feeders capable of ingesting a variety of food particles, including microalgae, bacteria and detrital particles (Moens & Vincx, 1997). The field data indicates that *Daptonema* prefers to live in surface sedimentary layers (Soetaert et al., 1995) avoiding the deeper layers with decreasing dissolved oxygen concentrations (Steyaert et al., 2003).

Group 3 contains aerobic microorganisms, as suggested by the presence of *Nitrospirales*, but also anaerobes as implied by the presence of possible sulfate reducing bacteria from the class *Deltaproteobacteria*, which use sulfate as electron acceptor in the absence of oxygen. Among nematodes, many OTUs of *Dorylaimopsis* are found. They are classified as epistrate feeders (Singh & Ingole, 2016), which scrape the microbiota from solid surfaces or feed on bacterial mucus threads (Giere, 2009). High abundances of epistrate feeders have been already reported in deep-sea habitats (Vincx et al., 1994).

Group 2 is characterized by the presence of potential sulfate reducers and can thus be defined by the presence of anaerobic bacteria. For nematodes, a large majority of the sequences are affiliated to *Terschellingia*, a cosmopolitan nematode, which tends to be very abundant in hypoxic or anoxic sediments (Steyaert et al., 2007) where chemosynthetic processes are important. *Terschellingia* is a microvore with a very small buccal cavity, enabling ingestion of only bacteria-size particles (Moens & Vincx, 1997). Stable isotopes analysis carried out at genus level indicated that *Terschellingia* relies predominantly or even exclusively on chemoautotrophic sulfide oxidizing and other oxidizing bacteria (Vafeiadou et al., 2014), but the finding of their co-occurrence with sulfate reducers may also suggest that they can prey on other type of bacteria.

Group 1 microorganisms are also possible anaerobes. Among nematode OTUs, *Halichoanolaimus* characterizes this group. It belongs to predators-omnivores (Vanhove, Arntz & Vincx, 1999; Singh & Ingole, 2016), which have diverse feeding habits and food sources. *Halichoanolaimus* feeds indiscriminately on others nematodes as well as bacteria and microorganisms. This nematode was already found in deep-sea anoxic sediments (Singh & Ingole, 2016).

In most of cases, the associations within groups could be linked to the ecological requirements of the nematodes and bacteria. For example, group 4 contains species able to cope with the presence of oxygen, including *Eubostrichus*, typically found in boundary zone of redox-potential-discontinuity, where sulfide and oxygen are simultaneously present (Schiemer, Novak & Ott, 1990; Berger, Urbancik & Ott, 1996). This nematode is remarkable because of its obligatory association with sulfide-oxidizing ectosymbiotic bacteria covering its entire body in an ordered and specific pattern (Ott et al., 1991; Polz, Harbison & Cavanaugh, 1999; Maurin et al., 2010). In this study, it is difficult to say whether the sulfide-oxidizing bacteria found in the group 4 are associated with the sediment environment or if they are the ectosymbionts colonizing nematode cuticle. The migration of the nematodes between sulfidic and oxic sediment layers may benefit the nematode-bacteria symbiosis (Schiemer, Novak & Ott, 1990). These sulfide-oxidizing bacteria may gain energy through sulfide oxidation and potentially recharge reduced sulfur storage under the form of elemental sulfur while the nematode stays in the sulfidic layer of the sediment and subsequently they gain energy from the oxidation of reduced sulfur when the nematode migrates into oxic layers (Schiemer, Novak & Ott, 1990; Ott et al., 1991). The migration behavior could also favor the storage of nitrate by bacteria in the oxic and suboxic layer as shown for free-living Beggiatoa bacteria (Preisler et al., 2007). The advantage chemosynthetic energy supply by its bacteria could explain the occurrence of this nematode under aerobic conditions. No free sulfide was detected in the sediment collected in associated cores down to 15 cm (Yücel, 2013). Nevertheless, black patches were observed and denoted the presence of sulfidic microniches as described by Stockdale, Davison & Zhang (2009). Sulfur cycling may be driven here by the export of plant debris such as wood, which was shown to sustain sulphate-reducing microbes producing sulfide (Charles et al., 2014; Kalenitchenko et al., 2017).

Furthermore, predatory nematodes are observed in all the groups. The importance of bacteria as food source or symbiotic associations for these nematodes remain poorly documented. The observation of predatory behavior on living nematodes (Moens & Vincx, 1997) suggests that they are mobile in the sediment (Schiemer, Novak & Ott, 1990). This observation rather underlines the need to take into account the existence of temporal aggregations in the nematode communities. It also highlights the fact that bacteria population in the sediment are not only structured by the vertical redox-gradient but also by interactions such as predation. Such biotic factors remain poorly understood and deserve further dedicated studies for a better understanding of the microbial functioning of deep-sea benthic ecosystem.

## Conclusion

The present study provides new data on the diversity of bacterial and nematodes communities in the Lacaze-Duthiers canyon, which represents a particularly heterogeneous deep-sea habitat over space and time. Specifically, high throughput sequencing approaches combined to MIC statistical analysis allowed us to identify, for the first time, at the same micro-scale, the community structures and the co-occurrence of nematodes and bacteria OTUs across the sediment depths. This detailed analysis reveals marked patterns of co-occurrences between the bacteria and nematodes in the sediment of the canyon. The result highlights the importance of small-scale sampling and combines diversity assessment for an accurate analysis of the variability and potential functions of deep-sea communities. The design of the present study may have missed some nematodes, and it did not allow to identify the environmental drivers lying behind the observed bacteria–nematodes associations, but the results provide useful data for future researches.

## Supplemental Information

10.7717/peerj.5396/supp-1 Supplemental Information 1 Fig. S1. A map of the area in the Gulf of Lion. A: map of the area in the Gulf of Lion showing the experiment site in the Lacaze-Duthiers canyon at the eastern end of the Pyrenean mountain (prepared using the GeoMapApp free software (www.geomapapp.org). B: location of sampling inside the Lacaze-Duthiers canyon (Adapted from Berné, S. & Satra, C. Gulf of Lions bathymetry (GIS shape files) 2003).

## Additional Information and Declarations

### Data Availability

The following information was supplied regarding data availability: Sequence data were deposited in the GenBank/EMBL/DDBJ short-read archive as submission ID: SRP127023 and SRP137819.
