# Supplementary material for: Co-occurring nematodes and bacteria in submarine canyon sediments
Source: PeerJ. 2018 Jul 31;6:e5396. doi: 10.7717/peerj.5396 (PMC6074754; doi:10.7717/peerj.5396)
Supplement: Supplemental Information 1 — A: map of the area in the Gulf of Lion showing the experiment site in the Lacaze-Duthiers canyon at the eastern end of the Pyrenean mountain (prepared using the GeoMapApp free software (www.geomapapp.org). B: location of sampling inside the Lacaze-Duthiers canyon (Adapted from Berné, S. & Satra, C. Gulf of Lions bathymetry (GIS shape files) 2003). [file peerj-06-5396-s001.pdf]

Co-occurring nematodes and bacteria in submarine canyon sediments.

Jadwiga Rzeznik-Orignac[1], Antoine Puisay[1,3,], Evelyne Derelle[2], Erwan Peru[1], Nadine Le Bris[1] and Pierre E. Galand[1]

[1]Sorbonne Université, CNRS, Laboratoire d'Ecogéochimie des Environnements Benthiques, LECOB, F-66650, Observatoire Océanologique, Banyuls, France

[2]Sorbonne Université, CNRS, Laboratoire de Biologie Intégrative des Organismes Marins BIOM, F-66650, Observatoire Océanologique, Banyuls, France

[3] PSL Research University: EPHE-UPVD-CNRS, Criobe, Laboratoire d'Excellence Corail,BP 1013, 98729 Papetoai, Moorea, French Polynesia

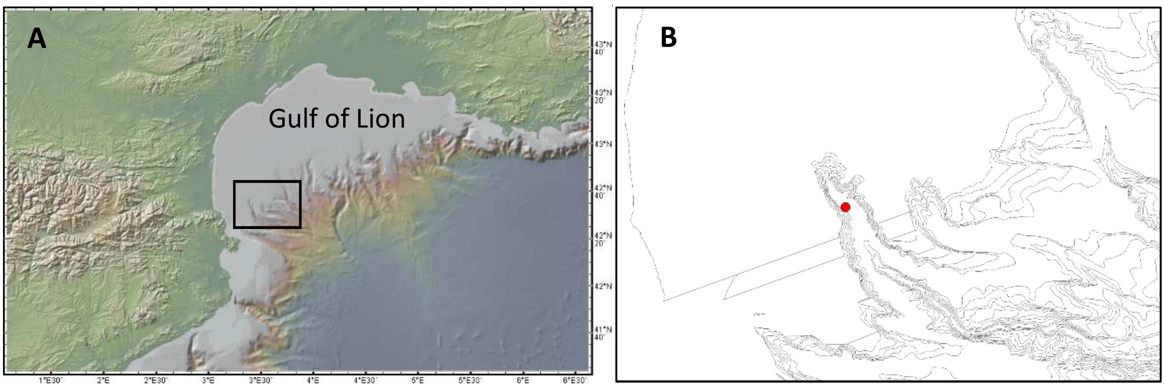

A: map of the area in the Gulf of Lion showing the experiment site in the Lacaze-Duthiers canyon at the eastern end of the Pyrenean mountain (prepared using the GeoMapApp free software (www.geomapapp.org).
B: location of sampling inside the Lacaze-Duthiers canyon (Adapted from Berné, S. & Satra, C. Gulf of Lions bathymetry (GIS shape files) 2003).

Berné, Serge; Satra, Catherine (2003): Gulf of Lions bathymetry (GIS shape files). PANGAEA, https://doi.org/10.1594/PANGAEA.89449
